# Structural Phylogenomics Reveals Gradual Evolutionary Replacement of Abiotic Chemistries by Protein Enzymes in Purine Metabolism

**DOI:** 10.1371/journal.pone.0059300

**Published:** 2013-03-13

**Authors:** Kelsey Caetano-Anollés, Gustavo Caetano-Anollés

**Affiliations:** 1 Evolutionary Bioinformatics Laboratory, Department of Crop Sciences, University of Illinois at Urbana-Champaign, Urbana, Illinois, United States of America; 2 Chicago School of Professional Psychology, Chicago, Illinois, United States of America; The Centre for Research and Technology, - Hellas, Greece

## Abstract

The origin of metabolism has been linked to abiotic chemistries that existed in our planet at the beginning of life. While plausible chemical pathways have been proposed, including the synthesis of nucleobases, ribose and ribonucleotides, the cooption of these reactions by modern enzymes remains shrouded in mystery. Here we study the emergence of purine metabolism. The ages of protein domains derived from a census of fold family structure in hundreds of genomes were mapped onto enzymes in metabolic diagrams. We find that the origin of the nucleotide interconversion pathway benefited most parsimoniously from the prebiotic formation of adenine nucleosides. In turn, pathways of nucleotide biosynthesis, catabolism and salvage originated ∼300 million years later by concerted enzymatic recruitments and gradual replacement of abiotic chemistries. Remarkably, this process led to the emergence of the fully enzymatic biosynthetic pathway ∼3 billion years ago, concurrently with the appearance of a functional ribosome. The simultaneous appearance of purine biosynthesis and the ribosome probably fulfilled the expanding matter-energy and processing needs of genomic information.

## Introduction

While the origin of life remains mysterious, the principle of continuity dictates that simple chemistries must precede complex biochemistry. Thus, the making of amino acids and nucleotides must precede polypeptides and nucleic acids. In other words, some chemistries of metabolism must be of prebiotic origin and should have been established prior to peptide bond biosynthesis and nucleic acid replication. Cellular metabolism is driven by a complex collection of enzymes, most made solely of proteins and few also involving nucleic acids (e.g., the RNase P complex) [1]. These enzymes are responsible for the catalysis of metabolite-transforming chemical reactions and for metabolite transport in cellular systems. Metabolites are small molecules that are products and intermediates of metabolic reactions. They are also part of large and highly interconnected networks in which the chemical output of one enzyme serves as input of one or may others. Protein enzymes in metabolic networks harbor active sites in one or more domains. These domains are compact three-dimensional (3D) arrangements of elements of secondary structure that are evolutionarily conserved [2]. Domains represent structural units in single or multi-domain proteins and are usually recurrent in metabolism. The modular use of domains in the protein world [3] suggests enzymatic functions and their associated protein structures have spread in evolution through recruitment, a common phenomenon in biology that occurs when a molecule, ensemble, repertoire, or a more complex system adapts an existing feature for a new purpose and within a different context. Since enzyme recruitment appears the main evolutionary driver of modern metabolism and is more common than *de novo* invention and enzyme and pathway duplication and specialization [1], [4], [5], it is therefore likely that this process played important roles during the very early replacement of prebiotic chemistries by biochemistries. Here we explore recruitment patterns in the most ancient metabolic network, purine metabolism [1], [5]. We put forth the hypothesis that enzyme recruitment in primordial cells benefitted from external prebiotic chemistries, which provided abundant raw materials and simplified the challenges of building efficient cellular metabolic systems from scratch. We test this hypothesis by asking if enzymatic recruitment history inferred from phylogenomic reconstruction is compatible with current understanding of prebiotic chemistry.

The principle of continuity also dictates that the thousands of protein domain structures of the cell must appear progressively in time – the time of origin of individual domains establishes their relative age. The MOLECULAR ANCESTRY NETWORK (MANET) database traces evolution of protein domains in biological networks and is therefore useful to study the history of modern metabolism [4]. The relative age of domains with molecular structures defined according to the STRUCTURAL CLASSIFICATION OF PROTEINS (SCOP) [6] are first obtained from phylogenetic trees, explicit statements of domain history built from a census of protein domain structure in the proteomes of hundreds to thousands of organisms that have been sequenced [2], [3], [7]–[10]. The age of domains is then mapped onto the enzymes and associated enzymatic functions that delimit network structure in illustrations of each and every subnetwork of metabolism [4]. This provides the means to find patterns of origin and evolution of metabolic functions. An initial analysis showed for example that metabolism originated in enzymes that participated in the interconversion of nucleotides in purine metabolism [5]. These enzymes harbor the P-loop hydrolase fold. Here we reexamine the origin of metabolism and the origin and evolution of the purine biosynthetic subnetwork.

Purine and pyrimidine nucleotides and their cognate bases can be produced *de novo* from simple precursors by biosynthetic pathways or by catabolic and salvage pathways that recycle nucleotide derivatives from turnover and degradation of nucleic acid compounds [11]. While most organisms can make purine and pyrimidine nucleotides from scratch the salvage pathways are the major source for synthesis of nucleic acids and enzyme cofactors of the cell. This is because nitrogenous bases do not generally serve as energy sources and cannot be used by pathways of energy conservation. The purine ring is biosynthesized in eleven enzymatic steps by the successive addition of nine atoms to ribose-5-phosphate. These atoms are contributed by carbon dioxide (C-6), aspartic acid (N-1), glutamine (N-3 and N-9), glycine (C-4, C-5 and N-7), and one-carbon derivatives of the tetrahydrofolate coenzyme (C-2, C-8). Pathways of nucleotide interconversion mediated by ATP-dependent kinases turn nucleoside monophosphates into energetically charged multiphosphate forms. Purine salvage involves phophorybosyltransferase enzymes that resynthesize nucleotides from their base constituents. Finally, purine degradation requires intracellular nucleotidases, nucleoside phorphorylases and deaminases to form inosine, xanthine and, by the action of an oxidase, uric acid [11]. In this paper we make use of MANET and the evolutionary ages of domains defined at the family level of structural complexity, which is close to protein sequence, to dissect the effects of recruitment and study the likely progression of enzymatic steps in the formation of the major purine metabolic pathways as these gradually replaced prebiotic chemistries of early Earth.

## Results

### Tracing the age of protein domains in enzymes of purine metabolism at fold family level reveals the emergence of central purine metabolic pathways

**Figure 1:**
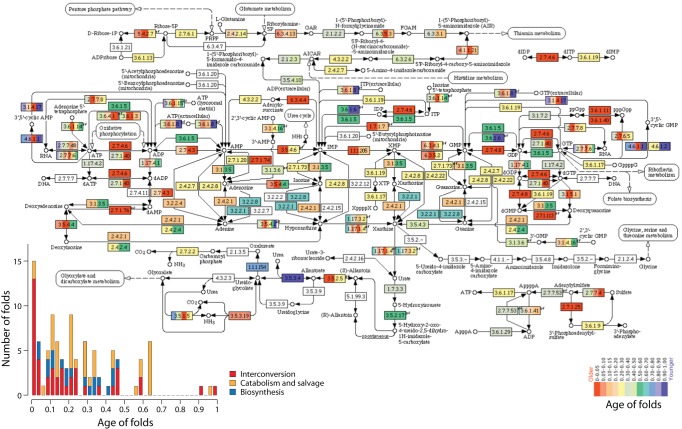
The purine metabolic subnetwork (NUC 00230) of MANET 2.0. Domain structures associated with individual enzymatic activities (described in EC nomenclature) are painted according to their age, in a scale of node distance (*nd_F_*) that ranges from 0 (the oldest enzymes) to 1 (the most recent).

The origin and evolution of the purine metabolic network was first explored using the MANET database [4]. MANET links protein taxonomies, metabolic networks and phylogenomic reconstructions derived from a genomic census of protein structure [4]. Taxonomies such as SCOP [6] and CATH [12] use protein domain building blocks as units of classification [13]. In SCOP, domains that are evolutionarily closely related at the sequence level are clustered into fold families (FFs). Domains belonging to different families that exhibit low sequence identities but that share structural and functional features suggesting a common origin are further unified into fold superfamilies (FSFs). Finally, FSFs sharing secondary structures that are similarly arranged and topologically connected are unified into protein folds. These folds sometimes have peripheral regions of secondary structure that add peripheral structural complexity to the central core fold architecture [14]. The two releases of MANET trace domain ages derived from structures defined at fold level of structural abstraction along metabolic pathways of the KYOTO ENCYCLOPEDIA OF GENES AND GENOMES (KEGG) [4]. Figure 1 shows the purine metabolism subnetwork (NUC 00230) of MANET 2.0 with ages of domains colored in a scale from the very oldest (*nd_FF_*  = 0; deep red) to the most recent (*nd_FF_*  = 1; deep blue). An old domain is here defined as one that had an earlier evolutionary origin when compared to a recent domain. The patchy distribution of colors in this subnetwork reflects the pervasive trend found in almost all 136 MANET subnetworks that confirms previous inferences that recruitment is the driving evolutionary force of metabolic expansion [15], [16]. More importantly, a quick examination of domain ancestries of enzymes in biosynthesis (BIO), catabolism and salvage (CAT), and regulatory interconversion pathways (INT) of purine metabolism suggest biosynthetic enzymes are relatively recent evolutionary additions to the enzymatic toolkit of this subnetwork (Figure 1). The most ancient enzymes (colored in deep red) are located in a horizontal transect of the subnetwork that is responsible for nucleotide interconversion, supporting previous evidence that metabolism originated in enzymes harboring the P-loop hydrolase fold and responsible for these kinase functions [5]. This same pattern was observed when analyzing NUC 00230 of MANET 1.0 (not shown).

MANET 1.0 and 2.0 use domains structures defined at fold level. This is problematic. Folds are ambiguously associated with molecular functions and cannot dissect recruitment patterns without additional information [1], [5]. In contrast, FFs are generally unambiguously linked to molecular functions and are more powerful in their ability to uncover the history of early biochemistry [17]. This power has been made evident in the study of the most ancient proteins [18], the rise of translation [19], the protein repertoire of the last universal common ancestor [9], the first amino acid biosynthetic pathways and the origin of aerobic metabolism and planet oxygenation [20], [21]. We therefore assigned ages to the FF domains of the purine metabolic subnetwork using phylogenomic trees reconstructed from an analysis of domain abundance in the proteomes of 989 organisms that have been completely sequenced (A989) [20] and a subset of 420 that are free-living (FL420) [19]. The rooted trees describe the evolution of 3,513 FFs (out of the 3,902 defined by SCOP 1.75). Evolutionary assumptions are made explicit in the Methods section. We present observations from the FL420 dataset but those of the A989 provide congruent results, which are not shown. We note that FL organisms are free from evolutionary constraints imposed by parasitic and obligate parasitic lifestyles. These lifestyles result in biases in the structure [8] and function [22] of their protein repertoires, a fact that becomes evident when reconstructing trees describing the evolution of their proteomes [8]–[10]. In contrast, the inclusion of non-FL organisms in datasets does not affect significantly the trees of domain structures and the conclusions of this study.

**Figure 2:**
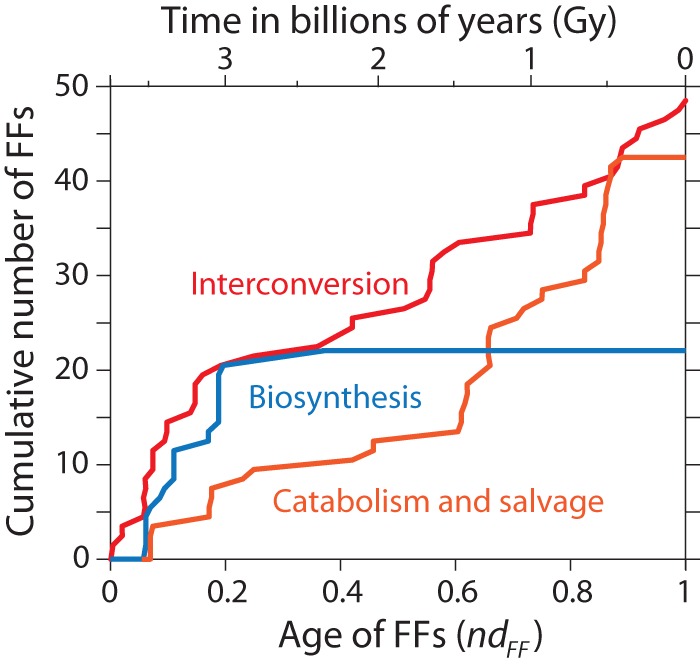
Evolutionary accumulation of protein domains at FF level of structural abstraction in the central pathways of purine metabolism.

**Figure 3:**
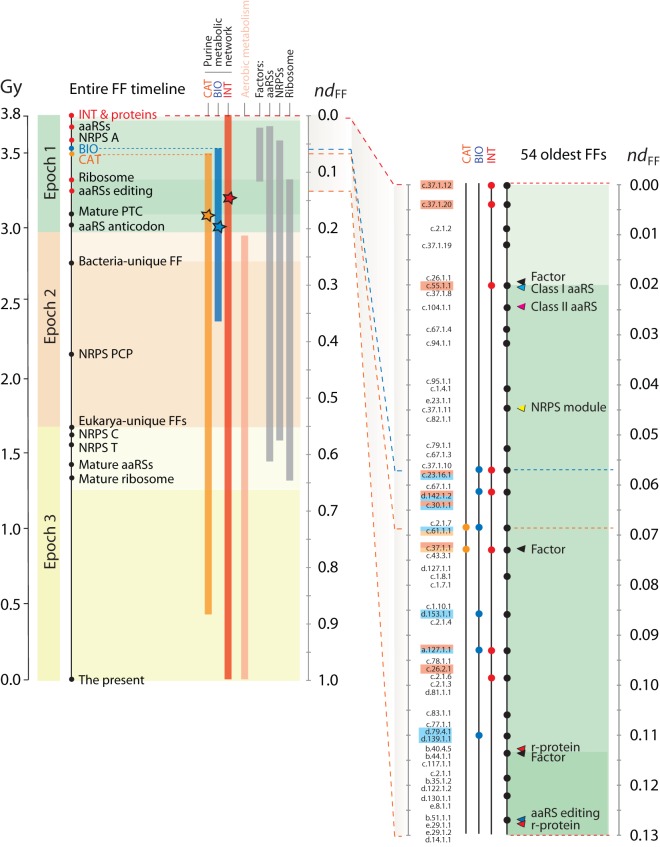
Timeline describing the evolution of FF domain structures and the evolution of main pathways of purine metabolism. The timeline was derived directly from the tree of FFs reconstructed from free-living organisms. Ages are given as node distances (*nd_FF_*) and geological time (Gy). Time flows from top to bottom. The three evolutionary epochs of the protein world defined by Wang et al. [8], “architectural diversification” (epoch 1), “superkingdom specification” (epoch 2), and “organismal diversification” (epoch 3) are overlapped to the timeline (colored with different shades). Landmark discoveries [18], [19] are identified with circles along the timeline. The inset below describes the evolution of the 54 most ancient FFs. Stars represent the fulfillment of a full repertoire of enzymes in a central pathway.

**Figure 4:**
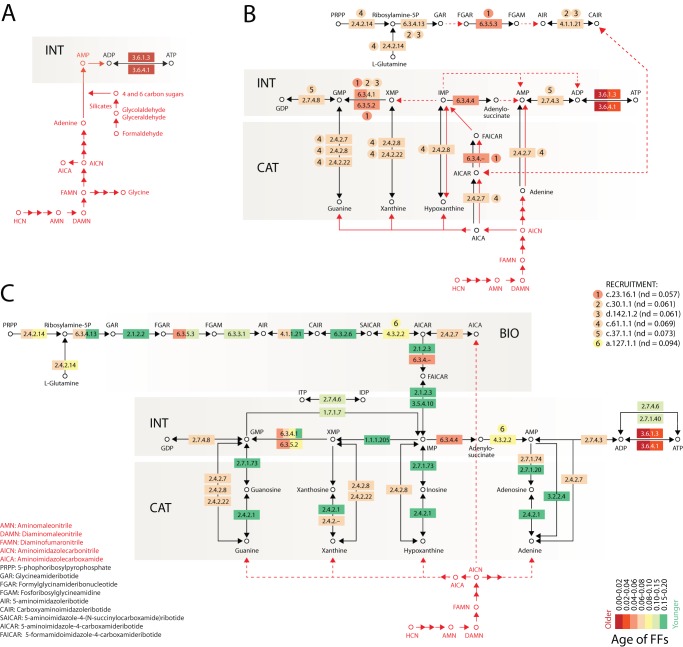
Early evolution of the purine metabolic network. A. Origin of nucleotide metabolism ∼3.8 Gy ago; *nd_FF_*  = 0). B. Emergence of the nucleotide interconversion (INT), catabolism and salvage (CAT) and biosynthetic (BIO) pathways ∼3.5 Gy ago (*nd_FF_*  = 0.061–0.073). C. Fully connected INT, BIO and CAT pathways ∼3 Gy ago (*nd_FF_*  = 0.187). Pathways mediated by prebiotic chemistries that are plausible and most parsimonious are depicted in red and enable the growth of the emergent protein enzyme-mediated pathways of purine metabolism by structural and functional innovation and piecemeal recruitment (recruited FFs are indicated with numbers). Unknown candidate or withering prebiotic pathways are indicated with dashed lines. We note that primordial reactions of the BIO pathway (top of metabolic diagram) in B could have been non-operational in the absence of suitable prebiotic chemistries until later in evolution. FF structures associated with individual enzymatic activities (described in EC nomenclature) are painted according to their age, in a scale of node distance (*nd_FF_*) that ranges from 0 (the oldest enzymes; ∼3.8 Gy ago) to 0.2 (∼3.0 Gy ago).

We examined the evolutionary accumulation of the enzymatic domains of the INT, BIO and CAT pathways of NUC 00230 along the evolutionary timeline of FF discovery (Table S1 and Figure S1) and recorded the time of the first appearance of these pathways (Figures 2 and 3). A quick examination shows that INT pathways originated at the start of the protein world (*nd_FF_*  = 0) and were only later followed by enzymes in BIO (*nd_FF_*  = 0.057) and then CAT (*nd_FF_*  = 0.069) pathways (Figure 4). Interestingly, while INT and CAT domains continued to accumulate essentially throughout the entire timeline, the establishment of the enzymatic domains of BIO occurred during a restricted time frame (*nd_FF_*  = 0.057–0.367) (Figure 3). This time frame is considerably smaller than timeframes of molecular accretion of ribosomal proteins in the ribosomal ensemble and the development of aminoacyl-tRNA synthetase (aaRS) and non-ribosomal protein synthase (NRPSs) specificities needed for the establishment of the genetic code, modern translation and specific non-ribosomal peptide assembly lines [18], [23]. These observations support the centrality of the purine metabolic network during the onset of early life.

### Placing the emergence of purine metabolic pathways in geological time scales

The age of FFs (*nd_FF_*) is provided in a relative zero-to-one scale, with *nd_FF_*  = 0 representing the origin of the protein world. However, we found that the ages of domain structures at fold or FSF levels show strong linear correlation with geological time scales measured in billions of years (Gy) [24]. This universal recurrence defines ‘molecular clocks’ of structural innovation, which were calibrated with multiple geological ages derived from the study of fossils and microfossils and geochemical, biochemical, and biomarker data (summarized in Methods). We note that the accuracies of the clocks are affected by the validity of each and every assumption used to support the molecular, physiological, paleontological and geochemical inferences of the supporting studies [24]. However, marker calibration can be revised by construction of updated or improved phylogenomic trees or by discovery, falsification, or confirmation of geochemical, biochemical, and biological markers. Moreover, new markers can be added to initial calibrations enhancing the precision of timescales. In this regard, the molecular clock of domain structures differs notably from standard ‘molecular clocks’, which are generally derived from the individual analysis of protein and nucleic acid sequences and are usually based on a single calibration point. These standard clocks suffer from variable ‘tick’ rates, bias from sequence saturation, uneven evolutionary rates within and between lineages, and limited precision [25], [26]. In contrast, molecular clocks of structures involve multiple calibration points and apply to the evolution of all know protein domains (not species), which encompass the entire history of life [24]. Since these clocks are not dependent on trees of species, the approach is independent of local rate variations in change of individual proteins or in branches of lineages.

We used the molecular clock of FSF domains to assign approximate geological ages to the origin of purine metabolic pathways, since the FFs of the most ancient enzymes were the most ancient in the corresponding FSFs. The nucleotide (energy) interconversion of the INT pathways appeared at the start of the protein world embodied in the ATPase domain-like FF (c.37.1.12) of ABC transporters, ∼3.8 Gy ago (Figure 3). In turn, the most ancient enzymatic domain of the BIO pathway, the class I glutamine amidotransferase (GAT) FF domain (c.23.16.1) of the formylglycinamide ribonucleotide amidotransferase [EC 6.3.5.3] enzyme, appeared later, ∼3.52 Gy ago, and the most ancient domain of the CAT pathway, the phophoribosyltransferase (PRTase) FF domain (c.61.1.1) of the xanthine-guanine phorphoribosyltransferase [EC 2.4.2.8] enzyme, appeared some few million years later, ∼3.5 Gy ago (Figure 3). These enzymes followed important structural innovations, including catalytic aminoacylating domains of aaRSs and NRPSs, both of which are known to be involved in peptide bond formation and are more ancient than ribosomal proteins and the ribosome [18], [23]. The enzymatic INT, BIO and CAT pathways probably emerged as a response to the demands of the primordial biosynthetic machinery, which at the time had the potential to aminoacylate primordial tRNA loops and produce small peptides [18]. Remarkably, all central enzymes of the biosynthetic pathway were already present ∼3 Gy ago, at *nd_FF_*  = 0.187 (Figure 3). This time coincides with the rise of the peptidyl transferase center (PTC) in the ribosome and a functional modern biosynthetic core of protein synthesis [9], [23] and sets up the stage for the appearance of the last universal common ancestor, aerobic metabolism and the rise of planetary oxygen, ∼2.9 Gy ago [9], [20], [24].

### Structural recruitment plays an important role in pathway formation

A group of six FFs crucially associate with the early expansion of the purine metabolic pathways, which is portrayed in a time series (Figure 4). The three oldest of these are the GAT FF (c.23.16.1; *nd_FF_*  = 0.057), the BC N-terminal domain-like FF (c.30.1.1; *nd_FF_*  = 0.061) and the BC ATP-binding domain-like FF (d.142.1.2; *nd_FF_*  = 0.061), which are shared by the GMP synthetases (EC 6.3.4.1 and EC 6.3.5.2) of the INT pathway and the AIR carboxylase (EC 4.1.1.21), FGAM synthetase (EC 6.3.5.3) and AICAR formyltransferase C-N ligases (EC 6.3.4.–) of BIO (Figure 4). The more recent PRTase FF (c.61.1.1; *nd_FF_*  = 0.069) is responsible for most salvage reactions of the CAT pathway and is shared with the BIO functions of AMP pyrophosphorylase (EC 2.4.2.7) and amidophosphoribosyltransferase (EC 2.4.2.14). In turn, the INT and BIO pathways share the L-aspartate/fumarase FF (a.127.1.1; *nd_FF_*  = 0.094), while the RecA-like protein like ATPase-domain FF (c.37.1.1; *nd_FF_*  = 0.073) enabled crucial GDP-GMP interconversions in the INT pathway.

The internal recruitment of structures as these appear in the timeline is responsible for the functional make up of the BIO and CAT pathways. Six out of the 11 reaction steps involved inter-pathway structural recruitment and another four involved intra-pathway recruitment. Only the SAICAR synthase FF (d.143.1.1; *nd_FF_*  = 0.188) of the enzyme EC 6.3.2.6 of the BIO pathway was not recruited from within the expanding purine metabolic network and appears to represent a unique and late development of purine metabolism. This is compatible with previous proposals that gene duplications mediated patchwork recruitments in the pathways of CAT [27]. Episodes of recruitment precede extensive domain accretion that is typical of the enzymes of purine metabolism and that continues to the present. Domain accretion is however absent during early evolution of nucleotide metabolism. The six FF structures that are recruited during early purine metabolic evolution are also wide contributors of structural scaffolds for other subnetworks. For example, the GAT FF c.23.16.1 of purine metabolism contributes to pyrimidine metabolism (NUC 00240), glutamate metabolism (AAC 00251), Phe, Tyr and Trp biosynthesis (AAC 00400), folate biosynthesis (COF 00790), and two component systems (MAP 02020) important for environmental information processing. According to MANET 2.0, each of the six FFs contributes structures to 3–10 subnetworks, and collectively to 20 subnetworks of metabolism, including pyrimidine metabolism. This attests to their functional versatility and ancestral nature.

### Prebiotic reactions mediate the evolutionary expansion of protein enzymes

We linked plausible prebiotic chemistries stemming from decades of origins of life research to the emerging pathways of protein enzymes, some of which mediate those same abiotic chemical reactions of early Earth (Figure 4). Systematic tracing of abiotic reactions that are most parsimonious with the recruitment-mediated expansion of enzymatic activities shows the slow replacement of prebiotic chemistries by enzyme-catalyzed counterparts, narrowing the possible and plausible prebiotic reactions that are needed to jumpstart modern metabolic routes.

#### (i) Tight origin of biochemistry and prebiotic chemistry

The enzymatic founders of nucleotide metabolism that appeared ∼3.8 Gy ago are embodied in primordial ATP phophohydrolases (EC 3.6.1.3 and EC 3.6.4.1) with the P-loop hydrolase fold structure. These enzymes enable ATP/ADP-mediated energy interconversion reactions. They include the first and second domain structures of the protein world, the ABC transporter ATPase domain-like (c.37.1.12; *nd_FF_*  = 0) and the AAA-ATPase domain (c.37.1.20; *nd_FF_*  = 0.004) FFs [18]. Both of these domain families contain a central interleaved fold design of a β-sheet flanked by α-helices. They also harbor a primordial bundle in their 3-dimensional fold that is probably a remnant of their ancient direct association with membranes [18]. We linked these ancient enzymes to prebiotic chemistries that provided them with a steady supply of adenosine phosphate cofactors needed for their mechanical membrane associated functions (Figure 4A). These chemistries produced a number of intermediates for the synthesis of nucleobases and ribose from HCN and formaldehyde precursors, respectively. We must emphasize that the evolutionary driver for the utilization of abiotically synthesized adenosine phosphate derivatives fulfills transport processes that would have been important for membrane stability and protocell persistence [18].

#### (ii) Quick development of purine metabolism with sequential replacement of prebiotic routes

Most central reactions of the INT and CAT pathways were already established 3.5 Gy ago (Figure 4B and C). In fact, domain structures were being recruited by a primordial BIO pathway at that time, or at most were soon after coopted, including GAT FFs responsible for the formation of 5-formamidoimidazole-4-carboxamideribotide (FAICAR) from 5-amino-4-imidazolecarboxamideribotide (AICAR) by formyltransferases. These recruitments or cooptions placed the enzymatic synthesis of nucleotides a step away from independence of abiotic reactions. In the 3.5–3.8 Gy ago interval, plausible prebiotic routes of production of hypoxanthine, xanthine and guanine [28], [29] were fully exploited to fulfill the needs of emerging GTPases that act as molecular switches [18] (Figure 3). Their presence enabled the appearance of GMP synthetases (EC 6.3.4.1 and EC 6.3.5.2) that mediate the conversion of XMP to GMP (Figure 4B). Note that pathways for carbohydrate and amino acid biosynthesis that were developing at that time [1] likely provided enzymatic intermediates for ribose and nucleotide synthesis before INT and CAT pathways were established. For example, the enzyme-mediated production of adenylo-succinate (EC 6.3.4.4) by the nitrogenase iron protein-like FF (c.37.1.10; *nd_FF_*  = 0.057) enabled a direct shortcut to fumarate in the citrate cycle (that was probably running in reverse [30]) via adenylosuccinate lyase (EC 4.3.2.2 with the L-aspartase/fumarase FF a.127.1.1; *nd_FF_*  = 0.094). Similarly, the phosphate binding protein-like FF (c.94.1.1; *nd_FF_*  = 0.033) enabled the biosynthesis of 5-phosphoribosylpyrophosphate (PRPP) precursors in the pentose phosphate pathway (CAR 00030) that could have been used for primordial semienzymatic biosynthetic steps. One of these, originally proposed by Becerra and Lazcano [27] as founder of the purine BIO pathway, was the condensation of PRPP with AICA to form 5-amino-4-imidazolecarboxamideribotide (AICAR), a step that was later overtaken by the activity of AMP pyrophosphorylase (EC 2.4.2.7) (Figure 4C). Indeed and as previously suggested [27], purine salvage pathways appeared only after phosphorylated sugar biosynthetic pathways made ribosides fully available. This suggests withering of main prebiotic routes in the expanding metabolic networks during this time (dashed lines in Figure 4C).

#### (iii) Fully connected INT, BIO and CAT pathways

A fully functional BIO pathway replaces the need for purine abiotic synthesis and is fully functional ∼3 Gy ago (Figure 4C), with a possible exception in abiotically synthesized AICA for AMP pyrophosphorylase activity. The eleven enzymes of the BIO pathway provide a steady supply of IMP by sequentially building the purine ring on a phophoribosyl scaffold: ammonia displaces pyrophosphate in PRPP, glycine, formate, ammonia, bicarbonate, and aspartate are then added sequentially, forming in each step a new C-N bond. In each step, an oxygen atom bound to carbon is phosphorylated and ammonia or an amine group displaces the phosphoryl group. Since consumption of ATP increases reaction irreversibility in the pathway, nucleotide interconversion levels of the INT pathway increase irreversibly and are expected to alter the dynamics of purine metabolism in evolution.

## Discussion

An ancient RNA world with ribozymes mediating the many enzymatic activities of metabolism is less parsimonious and incompatible with timelines of domain discovery and many other lines of evidence [31]. Proteins that bind RNA molecules appear very late in evolution (Figure 3), including ribosomal proteins [18], [19], and these co-evolve with expanding RNA structures in ribosomes [23], [32]. Phylogenies describing the evolution of molecular functions that are derived from Gene Ontology annotations support the late arrival of the RNA world [33]. These historical accounts are also compatible with rings of gene neighbors derived from an analysis of physical clustering of conserved genes in bacterial genomes [34]. While phylogenetic statements can be suspect on many grounds, congruence is one, if not the most, powerful statement in evolution. In this case, congruence strongly suggests that the RNA world is a late evolutionary development.

In the absence of early ribozymes, two possible scenarios can be used to explain the patchy age distribution of enzymes in metabolism: (1) Early proteins that are highly multifunctional could have provided enzymatic functions for missing steps in pathways, albeit with low efficiency [35], [36], or (2) Coexistence and possible coevolution of abiotic chemistry and modern biochemistry could have enabled the emergence of modern metabolic networks in a ‘semienzymatic’ process [37]. Both of these scenarios are feasible and may have occurred at different degrees along the evolutionary timeline. For example, the mapping of EC activities to enzyme domains in modern metabolism makes it highly unlikely that the primordial metabolic toolkit would have covered all necessary enzymatic steps required to jumpstart major biosynthetic pathways. In turn, enzyme promiscuity, a phenomenon that exists in modern enzymes and could have been favored in ancient proteins [38], would have fostered considerable exploration of diverse enzymatic functions. Substrate ambiguity for example enhances catalytic potential and chemical diversification, traits that would have benefitted cooption of primitive enzymes into varying roles. Since domain structure constrains accessibility to new enzymatic functions [1], [5], modern metabolism was poised to co-evolve with abiotic chemistries, slowly replacing and internalizing the primordial chemical reactions into the make-up of the primordial organism. As the planet changed and some chemical prebiotic cycles exhausted, so did plausible chemistries, making chemical internalization a historical contingency of metabolic evolution. Our structural phylogenomic study attempts to untangle the process by focusing on the very first metabolic network [5]. The results are remarkable. The timeline of enzyme domains in purine metabolism can be for the most part reconciled with plausible prebiotic reactions that would have provided the emergent cells and their associated metabolic networks with basic constituents for biochemical experimentation and growth. Recruitment appears central in pathway origination: proteins performing a particular function in one biological context are brought to perform a related or different function in a different context. The process however remained tightly linked to the abiotic reactions that were poised to be replaced, and was slow: purine biosynthesis required over 800 million years to complete.

Phylogenomic reconstructions show that the first proteins were P-loop hydrolase mechanoenzymes of nucleotide metabolism that appeared ∼3.8 Gy ago and were responsible for primordial energy interconversion reactions (Figure 4A). Since these reactions required abiotically synthesized ATP (or ATP analogs), we expect that nucleobases, ribose and glycosidic bond-mediated mechanisms of nucleobase attachment were prebiotically available at that time. In fact, prebiotic chemistry supports their existence:

- *Nucleoside constituents.* Nucleobases were likely produced by abiotic synthesis from HCN via its tetramer diaminomaleonitrile (DAMN) [28], [29]. DAMN has been shown to be an important intermediate for the formation of purines, pyrimidines and amino acids [39], [40] [reviewed in refs. 41,42]. In the presence of solar radiation DAMN efficiently converts to aminoimidazole carbonitrile (AICN), which produces aminoimidazole carboxamide (AICA) by partial hydrolysis [42], [43]. AICN enables the formation of adenine and AICA the formation of hypoxanthine, xanthine and guanine. Zubay [28], [29] proposed a prebiotic pathway for the non-enzymatic synthesis of inosine monophosphate (IMP) by sequential additions of HCN. Each step added a new C-N bond and ribose was added after the formation of the base. More recent experiments support prebiotic pathways from HCN that are catalyzed by minerals. In the presence of borate minerals, formamide (the hydrolysis product of HCN) forms purine and pyrimidine bases and carboxylic acids, the necessary constituents of nucleosides [44]. Ribose is particularly promoted in these reactions. The oldest borate minerals are magnesium borates, which can stabilize adenosine phosphate derivatives and sugars. Magnesium stabilizes diphosphate and triphosphate groups of nucleotides and promotes the condensation of orthophosphate to oligophosphates [45]. In turn, borate minerals stabilize pentose sugars by forming complexes with cis-hydoxyl groups, with furanose ring stabilization being maximal for ribose [46]. Under these circumstances, pentoses such as ribose can be formed by the formose reaction from formaldehyde and glycolaldehyde precursors [41]. Moreover, condensation of glycolaldehyde and formaldehyde in the presence of hydrated MgO in alkaline conditions renders 3 to 5-carbon sugars [47]. Similarly, clay silicates can mediate the formose reaction, control for sugar instabilities and provide homochirality, and when starting from glycolaldehyde and glyceraldehyde facilitate the formation of 4 and 6-carbon sugars [48]. Origins of life scenarios that involve atmosphere-generated HCN rained into alkaline aquifers generated by serpentinizing rocks are compatible with these and other experiments [46], making the abiotic synthesis of sugar backbones feasible.
- *Nucleoside formation.* The attachment of nucleobases to ribose via the glycosidic bond is troublesome. Purine nucleosides can be obtained by dry phase heating of purine bases with ribose in the presence of sea salts and magnesium [49]. Despite low yields (1–8%), this likely prebiotic reaction could have enabled the formation of AMP at levels compatible with those needed by energy interconversion. We note that according to the timeline of domain discovery, ATP formation was not used for RNA synthesis since the biosynthetic machinery was not in place 3.8 Gy ago (Figure 3). Instead, low yields could have still provided enough prebiotic throughputs for adenosine phosphate derivatives acting as peptide cofactors. In contrast, no similar prebiotic reaction is possible for pyrimidine nucleosides, especially because attaching pyrimidine bases to ribose is thermodynamically disfavored in water. We note that activated pyrimidine nucleotides can be formed in a short sequence of reactions proceeding through arabinose amino-oxazoline and anhydronucleoside intermediates [50]. While their prebiotic nature is plausible, these reactions have been criticized as solving the ‘water problem’ of the glycosidic bond at a cost of increasing the reactivity of carbohydrates to non-feasible levels [46]. Our domain timelines however solve the problem, since modern pyrimidine biosynthetic routes are recruited from early metabolic networks (including purine metabolism), making the need for prebiotic synthesis minimal in these more derived pathways (KCA and GCA, ms. in preparation).
- *Nucleotides.* Nucleotides can be readily synthesized from nucleosides by dry heating with for example ammonium dihydrogen phosphate [51], [52] or through reactions involving trimethaphosphates [41], [53]. The high yields of these reactions suggests these or similar pathways may have been abundant in primitive Earth. AMP to ATP abiotic reactions are therefore feasible and complete the synthesis of ADP for primordial ATP phophohydrolase activity.

During the 3.5–3.8 Gy time interval following the inception of primordial membrane-linked enzymes, the central α-β-α interleaved fold design typical of P-loop hydrolases was revisited in six new fold families necessary for enzymatic steps of the INT and CAT pathways (Figure 4B). Enzymatic recruitments during this time period were necessarily linked to prebiotic production of hypoxanthine, xanthine and guanine [28], [29]. Thus, the explosive expansion of metabolism [5] continued to interface with selected prebiotic chemistries during this time, while it populated enzymatic functions in other evolving metabolic pathways, including those of carbohydrate and amino acid biosynthesis [1]. Phylogenomic tracings show that enzymatic innovations in these other pathways likely provided enzymatic intermediates that could have benefited the evolution of the INT and CAT pathways, and vice versa, enzymes in these pathways could have been donors to other expanding metabolic pathways. The integration of phylogenomic tracings in the entire metabolic network structure will be able to untangle recruitment pathways during this crucial evolutionary period.

During the 3.0–3.5 Gy time interval, more efficient enzymatic steps of the INT and CAT pathways replaced the enzyme-coupled abiotic reactions and completed a fully functional biosynthetic pathway (Figure 4C). RNA was probably actively working as repository and messenger of genetic information ∼3 Gy ago. The PTC and processive regions of the ribosome had been already formed [23] and aaRSs had already accreted editing and anticodon-binding domains [19] (Figure 3). The emerging RNA world was fully embedded in the expanding world of proteins, and this required more effective synthesis of RNA components. In addition, the enzymes of fully developed nucleotide pathways continued to add new domains to their make up as time progressed. In fact, the INT and CAT pathways expanded their domain repertoire 3–4 fold (Figure 2). This expansion could have been triggered by economies of scale in the synthesis, interconversion and degradation of nucleotides.

Our phylogenomic study makes explicit a ‘metabolism-first’ model for origins of life exploration that takes into account the coexistence of abiotic and biotic metabolic reactions. The study attempts to merge prebiotic chemistry ‘nomothetic’ (bottom-up, universal and predictive) and evolutionary genomic ‘ideographic’ (top-down, historical and retrodictive) frameworks. In combination, the discovery operations (*sensu* [54]) of these frameworks, which link theory and observation, put forth historical scenarios that are falsifiable and explain the actual progression of enzymatic innovation in metabolism. We note that ideographic scenarios can maximize explanatory power and severity of test by for example encompassing larger datasets (e.g., genomes, structures, molecular functions, plausible chemistries), perfecting classifications (structures, enzymatic activities), eliminating sampling biases, improving phylogenomic reconstructions, and developing better models of phylogenetic character change. For example, the evolutionary patterns we revealed rely on the protein databases analyzed and can be affected by under or overrepresentation of sequences and structures, incorrect structural assignments, and effects of genome sampling. For example, the contents of structural repositories such as the Protein Data Bank (PDB) and SCOP, or genomic, functional and network information in KEGG, are biased by research preferences for targets and organisms, methodological constraints on crystallography, NMR spectroscopy and biochemistry, and proper knowledge integration and ontological definition. Similarly, structural definitions in molecular classification are always problematic and generally assume a discrete molecular world, which by definition must be considered to be a rough approximation. Finally, phylogenetic trees are evolutionary statements that are derived from molecules that are modern. Consequently, any claims from these trees are necessarily linked to the design and structure of extant molecules and not to those of their predecessors. While we trust that modern molecules have preserved features that are informative of their predecessors, the extent of structural and functional canalization must be put to the test [31]. Overcoming some of these limitations within the confines of our discovery framework should only enhance the confidence and explanatory power of phylogenomic statements.

## Methods

### Phylogenomic analysis

We reevaluated the history of purine metabolism using two versions of the MANET database (http://MANET.illinois.edu). MANET 1.0 (July 2006) provides 1,255 (out of 4,362) structural assignments (6,552/12,778 PDB entries) to enzyme activities in a total of 133 (out of 137) subnetworks of KEGG. Conversely, MANET 2.0 (July 2007) provides 1,405 (out of 4,673) structural assignments (7,238/18,744 PDB entries) to enzyme activities in 136 (out of 221) subnetworks. Both versions trace the ages of protein folds in the enzymes of metabolism, with ages derived from a structural phylogenomic census of 784 folds defined by SCOP 1.67 (May 2004) in the proteomes of 174 organisms with genomes that have been completely sequenced (19 Archaea, 117 Bacteria and 36 Eukarya) [4]. In order to diminish ambiguous structural-to-functional assignments and in anticipation to an updated release of MANET that will trace domain ages defined at the lower FSF and FF levels of structural abstraction in SCOP in all subnetworks of metabolism, a timeline of domain discovery derived from phylogenomic trees of domain structures defined at FF level was used to trace the evolution of subnetworks with curated entries of NUC 00230 from MANET 2.0. In this case, structural domains corresponding to 3,513 FFs defined by SCOP 1.75 (Feb 2009) were assigned to the proteomes of 989 cellular organisms (76 Archaea, 656 Bacteria, and 257 Eukarya) [20]. This structural genomic census uses the iterative SEQUENCE ALIGNMENT AND MODELING (SAM) method to scan genomic sequences (with probability cutoffs *E* of 10^−4^) against a library of advanced linear hidden Markov models (HMMs) of structural recognition in SUPERFAMILY [55]. The census produced a data matrix with columns representing proteomes (phylogenetic characters) and rows representing FFs (phylogenetic taxa). This matrix was used to build rooted phylogenetic trees of FF domain structures using the maximum parsimony (MP) method in PAUP* version 4.0b10 [56] and a combined parsimony ratchet (PR) and iterative search approach [3] to facilitate tree reconstruction and avoid the risk for optimal trees being trapped in suboptimal regions of tree space. A single MP reconstruction from the entire dataset (A989) was retained following 300 ratchet iterations (10×30 chains) with 1,000 replicates of random taxon addition, tree bisection reconnection (TBR) branch swapping, and maxtrees unrestricted [20]. A single MP tree was also reconstructed from the subset of organisms with free-living lifestyles (FL420) in A989 [19]. The main assumption of the phylogenomic approach is that protein domain structure holds deep phylogenetic signal, with domain age being in general proportional to domain abundance in proteomes. At high levels of structural complexity evolutionary change occurs at extraordinarily slow pace. A new fold family may take hundreds of thousands of years to materialize in sequence space while new sequences develop in less than microseconds [2]. For example, a recent comparative analysis of aligned structures and sequences showed structures were 3–10 times more conserved than sequences [57]. The model of phylogenetic character transformation invokes polarization of change towards increases in genomic abundance of domain structures. Operationally, character states in the data matrix were polarized from ‘N’ to ‘0’ using the ANCSTATES command of PAUP*, where ‘N’ indicates the plesiomorphic (ancestral) state. This ‘evolutionary arrow’ induces rooted trees without resorting to outgroup hypotheses, but does not constrain gains or losses in individual lineages. For example, the method has revealed important reductive evolutionary trends, especially in Archaea [10]. The biological basis for global increases in domain abundance is the existence of processes of gene duplication, amplification and rearrangement in genomes (e.g. [58]) that drive molecular innovation. Abundance should therefore be considered a natural evolving ‘heritable’ trait. This natural trait produces marked imbalance in trees, which we can measure as departures from uniform (Yule) and random speciation models [24]. Consequently, the relative age of domain structure (*nd_FF_*) can be calculated directly from rooted phylogenomic trees using a PERL script that counts the number of nodes from the ancestral structure at the base of the tree to each leaf and provides it in a relative zero-to-one scale. We stress that the evolutionary model applies to the world of proteins, does not constrain the spread of domain structures in proteomes (gain, loss, and convergent processes), and is agnostic about how domain growth occurs in proteomic lineages (discussed in [9], [10]). These and other properties allow construction of evolutionary timelines and enable a protein-centric view that links enzymatic activities to domain structures. This view can systematically track recruitment patterns and enzymatic cooptions throughout biological history [4], [5], [59]. The use of molecular structure and abundance in phylogenomic analysis offers numerous benefits over traditional methods [60]. For example, the use of multistate characters of genomic abundance mitigates the phylogenetic problems of alignment, taxon sampling, tree imbalance, phylogenetic inapplicables, and the serious problem of structure-induced violation of character independence that plagues phylogenetic sequence analysis. In particular, phylogenomic reconstruction is robust against uneven sampling of genomes across the three superkingdoms [9].

### Structural nomenclature

Concise classification strings (*ccs*) defined SCOP domains at fold, FSF and FF levels in SCOP. For example, c.23.16.1 represents the structure of the amidotransferase domain of the formylglycinamide ribonucleotide amidotransferase from *Salmonella typhimurium* (PDB 1T3T; Uniprot ID P74881) [61], a crucial enzyme of purine biosynthesis, where c represents the protein class, 23 the F, 16 the FSF, and 1 the FF. *ccs* were used to identify taxa in trees and label structures in diagrams.

### Structural assignment update and domain age assignments

HMM analysis of sequences of enzymes with no PDB models was conducted again on the set of purine metabolism enzymes. Since NUC 00230 had 83% structural coverage in enzymatic entries (83%), only few new structures were found associated with enzyme activities (4 linked to EC 1.17.4.2, EC 1.7.3.3, EC 2.7.7.7, EC 2.7.7.53 and EC 3.6.1.29) and none were relevant to the pathways analyzed in this study. Only SCOP entries for EC 3.5.4.2 sequences had to be revised (c.1.9.18 was confirmed with a crystallographic model, 2ICS). When assigning FF age, some FFs did not have *nd_FF_* values for the F420 set. In those few cases the age was assigned using the A989 dataset (a.35.1.2, c.1.9.14, c.1.9.18, c.97.1.4, a.5.1.1, h1.5.1 and h.1.26.1) or by using the largest *nd_FF_* value in the set of FF domains belonging to an FSF (b.92.1.5, b.92.1.7 and b.92.1.9). We also found that some domains without fold assignments in MANET had FF assignments (e.g. d.284.1.2 of EC 3.5.4.2) and could be incorporated in the analysis at this level of abstraction.

### Assigning geological ages to domains

The molecular clocks for protein domains at fold (t = –3.802 *nd* +3.814) and FSF (t = –3.831 *nd* +3.628) levels [24] were used to calculate the geological ages of selected families (in Gy), provided that FFs were the most ancient in each group. Calibrations revealed a significant linear correlation (P<0.0001) between the age of fold and FSF structures and geological time. They included a boundary fold associated with the earliest evidence of biological activity (ion microprobe analysis and isotopic composition of carbonaceous inclusions in 3.8 Gy-old banded iron rock formations), a fold linked to the biosynthesis of porphyrins (spectroscopic identification of vanadyl-porphyrin complexes in carbonaceous matter embedded in 3.49 Gy-old polycrystalline rocks), folds of enzymes of nitrogen assimilation (with ages inferred mostly from biogeochemical evidence), folds linked to lipid biomarkers such as hopanoids and biphytanes recovered from kerogen, bitumens and hydrocarbons, folds that are markers of bacterial and eukaryotic diversification episodes with times established from microfossil evidence (e.g., unicellular cyanobacterial coccoids in 1.9 Gy-old tidal sedimentary rock, acritarchs in 1.5 Gy-old rocks of the Roper Group of Northern Australia) integrated with molecular, physiological, paleontological and geochemical data, folds linked to biological processes and lineages (e.g., biosynthesis of flavonoids and red algae, hemocyanins and mollusks), and finally, present day boundary folds. Details and supporting references were previously described [24].

## Supporting Information

Figure S1 **Evolution of major pathways of the purine metabolic subnetwork (NUC 00230).** Domain structures defined at the FF level of structural abstraction that are associated with individual enzymatic activities (described in EC nomenclature) are painted according to their age, in a scale of node distance (*nd_FF_*) that ranges from 0 (the oldest enzymes) to 1 (the most recent).
(EPS)

Table S1 **Enzyme activities and associated FF assignments in purine metabolic pathways.**
(PDF)
