# Supplementary material for: Structural Phylogenomics Reveals Gradual Evolutionary Replacement of Abiotic Chemistries by Protein Enzymes in Purine Metabolism
Source: PLoS One. 2013 Mar 13;8(3):e59300. doi: 10.1371/journal.pone.0059300 (PMC3596326; doi:10.1371/journal.pone.0059300)
Supplement: Table S1 — Enzyme activities and associated FF assignments in purine metabolic pathways. (PDF) [file pone.0059300.s002.pdf]

Table S1 Enzyme activities and associated FF assignments in purine metabolic pathways

Pathway	Enzyme	FF domain	nd_{FF}
INT	EC 3.6.4.1	c.37.1.12	0.000
INT	EC 3.6.1.3	c.37.1.20	0.004
INT	EC 3.6.1.3	c.55.1.1	0.020
INT	EC 6.3.4.1	c.23.16.1	0.057
INT	EC 6.3.4.4	c.37.1.10	0.057
INT	EC 6.3.5.2	c.23.16.1	0.057
INT	EC 6.3.4.1	d.142.1.2	0.061
INT	EC 6.3.4.1	c.30.1.1	0.061
INT	EC 2.7.4.8	c.37.1.1	0.073
INT	EC 2.7.4.3	c.37.1.1	0.073
INT	EC 4.3.2.2	a.127.1.1	0.094
INT	EC 6.3.4.1	c.26.2.1	0.098
INT	EC 6.3.5.2	c.26.2.1	0.098
INT	EC 2.7.4.6	d.58.6.1	0.139
INT	EC 2.7.1.40	c.1.12.1	0.147
INT	EC 1.7.1.7	c.1.5.1	0.147
INT	EC 1.1.1.205	c.1.5.1	0.147
INT	EC 6.3.4.1	b.84.1	0.160
INT	EC 3.6.1.19	c.51.4.1	0.192
INT	EC 3.5.4.6	c.1.9.1	0.249
INT	EC 3.6.4.1	c.37.1.2	0.360
INT	EC 6.3.4.1	d.104.1.2	0.390
INT	EC 6.3.5.2	d.52.2.1	0.420
INT	EC 6.3.4.1	d.52.2	0.420
INT	EC 3.6.1.3	b.40.4.2	0.510
INT	EC 3.6.1.3	a.60.2.1	0.547
INT	EC 3.6.1.3	a.4.5.11	0.555
INT	EC 2.7.4.3	g.41.2.1	0.555
INT	EC 3.6.1.6	b.2.3	0.559
INT	EC 3.6.4.1	b.34.3.1	0.560
INT	EC 3.5.4.6	c.37.1.14	0.580
INT	EC 3.6.1.3	a.5.1.1	0.606
INT	EC 3.6.1.14	h.4.8.1	0.736
INT	EC 3.6.1.17	d.113.1.1	0.718
INT	EC 1.7.1.7	d.37.1	0.73
INT	EC 1.1.1.205	d.37.1.1	0.731
INT	EC 3.6.1.6	b.67.3	0.735
INT	EC 3.6.1.5	b.67.3.1	0.735
INT	EC 3.6.1.14	b.93.1.1	0.735
INT	EC 3.6.1.14	a.137.8.1	0.735
INT	EC 2.7.1.40	c.49.1.1	0.824
INT	EC 2.7.1.40	b.58.1.1	0.824
INT	EC 3.6.1.8	b.82.7.1	0.870
INT	EC 3.6.1.8	f.33.1.1	0.882
INT	EC 3.6.1.8	c.108.1.7	0.886

INT	EC 3.6.4.1	h.1.26.1	0.890
INT	EC 3.6.1.8	d.220.1.1	0.914
INT	EC 3.6.4.1	h.1.5.1	0.920
INT	EC 3.6.4.1	b.1.2.1	0.963
INT	EC 3.6.4.1	b.69.4.1	0.988
INT	EC 3.6.4.1	c.37.1.9	1.000
BIO	EC 6.3.5.3	c.23.16.1	0.057
BIO	EC 6.3.4._	c.23.16.1	0.057
BIO	EC 6.3.4.13	d.142.1.2	0.061
BIO	EC 4.1.1.21	d.142.1.2	0.061
BIO	EC 6.3.4.13	c.30.1.1	0.061
BIO	EC 4.1.1.21	c.30.1.1	0.061
BIO	EC 2.4.2.14	c.61.1.1	0.069
BIO	EC 2.4.2.14	d.153.1.1	0.086
BIO	EC 4.3.2.2	a.127.1.1	0.094
BIO	EC 6.3.5.3	d.79.4.1	0.110
BIO	EC 6.3.3.1	d.79.4.1	0.110
BIO	EC 6.3.5.3	d.139.1.1	0.110
BIO	EC 6.3.3.1	d.139.1.1	0.110
BIO	EC 3.5.4.10	c.97.1.4	0.170
BIO	EC 2.1.2.3	c.97.1.4	0.170
BIO	EC 6.3.4.13	b.84.2.1	0.188
BIO	EC 4.1.1.21	b.84.2.1	0.188
BIO	EC 3.5.4.10	c.24.1.3	0.188
BIO	EC 2.1.2.3	c.24.1.3	0.188
BIO	EC 2.1.2.2	c.65.1.1	0.188
BIO	EC 6.3.2.6	d.143.1.1	0.188
BIO	EC 4.1.1.21	c.23.8.1	0.196
BIO	EC 6.3.5.3	d.284.1.2	0.347
BIO	EC 6.3.5.3	a.5.10.1	0.367
CAT	EC 2.4.2.8	c.61.1.1	0.069
CAT	EC 2.4.2.7	c.61.1.1	0.069
CAT	EC 2.4.2.22	c.61.1.1	0.069
CAT	EC 2.7.1.74	c.37.1.1	0.073
CAT	EC 3.2.2.4	c.56.2.1	0.171
CAT	EC 2.4.2.1	c.56.2.1	0.171
CAT	EC 2.7.1.73	c.72.1.1	0.176
CAT	EC 2.7.1.20	c.72.1.1	0.176
CAT	EC 1.17.1.4	c.68.1.13	0.230
CAT	EC 3.5.4.4	c.1.9.1	0.249
CAT	EC 3.1.3.6	c.106.1	0.420
CAT	EC 3.1.3.5	1.17.3.2	0.457
CAT	EC 3.1.3.5	d.114.1.1	0.457
CAT	EC 3.5.4.2	b.92.1.1	0.604
CAT	EC 3.5.4.2	c.1.9.2	0.610
CAT	EC 3.5.4.2	a.35.1.2	0.610
CAT	EC 3.5.4.2	e.7.1.2	0.616
CAT	EC 3.5.4.2	c.1.9.9	0.620
CAT	EC 3.5.4.2	c.1.9.14	0.620

CAT	EC 3.5.4.2	c.1.9.18	0.640
CAT	EC 3.5.4.2	b.92.1.3	0.660
CAT	EC 3.5.4.2	b.92.1.5	0.657
CAT	EC 3.5.4.2	b.92.1.7	0.657
CAT	EC 3.5.4.2	b.92.1.9	0.657
CAT	EC 3.5.4.3	c.97.1.2	0.661
CAT	EC 3.1.3.5	c.108.1.8	0.706
CAT	EC 1.17.3.2	d.15.4.2	0.750
CAT	EC 1.17.1.4	d.15.4.2	0.751
CAT	EC 3.2.2.8	c.70.1.1	0.824
CAT	EC 3.2.2.1	c.70.1.1	0.824
CAT	EC 3.5.4.4	c.69.1.24	0.848
CAT	EC 3.5.4.4	b.70.3.1	0.848
CAT	EC 1.17.1.4	d.87.2.1	0.853
CAT	EC 1.17.3.2	d.87.2.1	0.853
CAT	EC 1.17.1.4	a.56.1.1	0.857
CAT	EC 1.17.3.2	a.56.1.1	0.857
CAT	EC 1.17.3.2	d.41.1.1	0.861
CAT	EC 1.17.1.4	d.41.1.1	0.861
CAT	EC 1.17.3.2	d.145.1.3	0.865
CAT	EC 1.17.1.4	d.145.1.3	0.870
CAT	EC 1.17.3.2	d.133.1.1	0.869
CAT	EC 3.5.4.2	c.1.9.6	0.890
